# Supplementary material for: NALCN/Cch1 channelosome subunits originated in early eukaryotes
Source: J Gen Physiol. 2025 Sep 5;157(6):e202413636. doi: 10.1085/jgp.202413636 (PMC12412391; doi:10.1085/jgp.202413636)
Supplement: Data S1 — provides protein sequences and a global alignment of the UNC80 homologues from human and the fungal species R. delemar. [file jgp_202413636_datas1.docx]

**UNC80**

>KAG1050648.1 hypothetical protein G6F43_007098 [Rhizopus delemar]

MSQEDQNDLVKNYKEESSRDATLDKQYKKKTKHFSAWDKLRENVSEYSSDTLYHATSDPSRPNMSKMSSPMHKKQMYNPFLRNQHTTTAENPLHDKINLTNTTLVAAQAAGMSSFVRNFKSKVGAGRHNGAIYATSTAVQQDIYRLERDLEKLLSHMNNRSQNASFTESESSNHNTVYSSDSLALNNFSVKSASKKTIFHRHSQAVLDPSTQHNEKHLDVTRISANITSIMDIVNKHKSATRLPFTSEILAVLSIPFDRKPQKTEDCIQALDAFDHIRSRFTLLESSEHFEQVLFCCSILGSSGIDLKEKILDILKKLVEPCFTYPNYLPSTPTAFHALAYALATSFAKQTQNIELQEKKTTYDLVRSGILSLLDNLANGKLISVQGDQWTTYFHTTQAASASLAKICMVESLCKCLMVSICRQTSFHERNAELFLNNLIQKDQDIPTLLELLQRYWTEPDTEILPAYFKSLGLLTELASEISLALSIEKLKSSAIPLLFNFILEKSAPSNVYNYLNQAISGKIQKSVIFNIITMLLCFLSVDLLTAPSRSAPSSNTPRTPPNLTPQSSLNDMFISSPVLDDSGSEAPHIIVSPEEAFLSQLLETAKAYLIEMWNYGYKSHICKCIEIMLQDTSENQLAHTYQNLLFHIDIAIGEEIAKVTIPILFKRILDTSPAPTPDFCSMLYQLSQRFRPLFYKPVVSCVASNDNDKVASLLKLMTSLRQYLSGVQFWMQDAEMINVLLLSDVGPKKLQRETSSLQQNHTSSTNLLTWGSTTLGQCIIATELVWVLKELREKQKDPDRNMEEDEVAKKFLIDLERRLALFLTAKEKKVLVPLPLRIILCNLFADARFFCYTTHRPGWLTCAINWATQPRTNDIGSQAISETFLDENLSETGFCSNDMSLPVLNANHIEEALIMFQRMQNTYLDVVEYFRDESSTSHGDSHGTRQAASSLLFSNTQMTENLKYGRRHLAETMYPINQSSSAALNLNPPEFDHLENNEGESNSIMLDLAKHKFDGIEEINQDPFGSVFSLLAAVYSTLSSHELGRLLKPLWKTYMDDKKAASFTPAAFLLMECGEKIPKLTTEMFSQALQRLNILNQSYIQISSRNKPFRGDGGAFATPFLPTDLGSNEFSLDEPRWMSKLKNTSNLPIELKRQIQELGWNEDDNAEEQEAFKKALTPLGLLPSLFLKDDNEDTNDADNPNIVVIRERGKIVDISKIITRRKRTATVHSFTAMFLTTVDLLMDDFAGVNNVLRELIEQFLKDDPALFLRAFLNNLGKHKADSLKDTLTRIRYIISMQRKLPPGLTHILFNYLAGMLKWIVRENQPDGLKFMTLVHPILAELTLSTNDLSVRDLRKNKIEHLLVSTGKFWFTHEQPADMFPRYLSNEQHSFAVLNIPQQIFFVACLRISHIQFLINYLARFPREVYAVKKTLQDYEPMPVPGSKWETRQLMEKAYYPDLSRRKNRQSNHKQEPLDTEDSQQHFDKEMLSLMRARIWLRFIDVLLNGLNKNYNDHDELERILQGVNTIMIEHSHDFGIIGQILILYTRMVTKFKRLFKSNRGYIIFLPALFKVFCESEDNPQITSAITFAWCRFYAVHEEAFVFQMLGALVPNILMIYGQSDEVGHWMIENLFKLMQAMDNPPHLGSAPDTLGLQLQVELDDHERSIQDRIDTASSQVSSALSVSIFKPLARGVTAPISQMEISTYNNRPFKLEDFIKLFLTVIAYDPGSLRAEQFVKVFTFLIPFFLEQSHLKSMMHEGASALVNVFVKFSKSVKSVVTTNSHTTSGEYQGISSSDSRLPGSNNAANTGPKAESAQNAYGKQWQQNDRFTIKQEFITLVHKYMKHGGTLSEVSHEKMAQIIRIVLRDYRNIKGLTVKTDWIKDYLVVSMYSMVDTRNYTKSLKKVLSEIYAQYRVQWKTVDASDLYEGLAIMLEQGQGKAIMMNDLAGLVKEKFVSFGLTMATLTDWQNNTKGQVKFCNSLVRLIIAILENSSQDVFAEIERQVPSVGLIGRVVIPLCLQYNLQWEYSTVGISSTSELNSANNWTRLLNFIAGVCSHTSLLKSKSSGFSLSQFTTAMGQTNIEDEVDMSDFPEAKDGKQTPQSVAVLFSLSLIAIKIILIRSHNTFNDLKGSWSQIAFFIRNTLVFGQTLKLLKAKVGRSDSYNLASPSDVLSPGMPSPSFFSPLGLSSPGASPYLSSNNPSLSPDLRANFSSNAPLGTGTIYDFTTWRFLEFVVCFRTPLFVLLKDFIHEKLGQMGPNVGSYRNSLYSPRASFNFPQNENTRWKSWGAEHSSVNTSGDGQSNANASAHSIVIPKLNIEDTGGVDAETLPTFGLGLHKSNSSASPGHVSHSPTGYSETDSSSPRSPTTNPFYKQQQHMSPGADVSKENNLHLLQAETITSVVNVQVAVGFKPALPWITMDSRDRSKPWNKKEAVARIANEWQLLLQLSTETDPLVNKANNNGSSANISASPLV

>Homo_sapiens_NP_115893.1

MVKRKSSEGQEQDGGRGIPLPIQTFLWRQTSAFLRPKLGKQYEASCVSFERVLVENKLHGLSPALSEAIQSISRWELVQAALPHVLHCTATLLSNRNKLGHQDKLGVAETKLLHTLHWMLLEAPQDCNNERFGGTDRGSSWGGSSSAFIHQVENQGSPGQPCQSSSNDEEENNRRKIFQNSMATVELFVFLFAPLVHRIKESDLTFRLASGLVIWQPMWEHRQPGVSGFTALVKPIRNIITAKRSSPINSQSRTCESPNQDARHLEGLQVVCETFQSDSISPKATISGCHRGNSFDGSLSSQTSQERGPSHSRASLVIPPCQRSRYATYFDVAVLRCLLQPHWSEEGTQWSLMYYLQRLRHMLEEKPEKPPEPDIPLLPRPRSSSMVAAAPSLVNTHKTQDLTMKCNEEEKSLSSEAFSKVSLTNLRRSAVPDLSSDLGMNIFKKFKSRKEDRERKGSIPFHHTGKRRPRRMGVPFLLHEDHLDVSPTRSTFSFGSFSGLGEDRRGIEKGGWQTTILGKLTRRGSSDAATEMESLSARHSHSHHTLVSDLPDPSNSHGENTVKEVRSQISTITVATFNTTLASFNVGYADFFNEHMRKLCNQVPIPEMPHEPLACANLPRSLTDSCINYSYLEDTEHIDGTNNFVHKNGMLDLSVVLKAVYLVLNHDISSRICDVALNIVECLLQLGVVPCVEKNRKKSENKENETLEKRPSEGAFQFKGVSGSSTCGFGGPAVSGAGDGGGEEGGGGDGGGGGGDGGGGGGGGGGPYEKNDKNQEKDESTPVSNHRLALTMLIKIVKSLGCAYGCGEGHRGLSGDRLRHQVFRENAQNCLTKLYKLDKMQFRQTMRDYVNKDSLNNVVDFLHALLGFCMEPVTDNKAGFGNNFTTVDNKSTAQNVEGIIVSAMFKSLITRCASTTHELHSPENLGLYCDIRQLVQFIKEAHGNVFRRVALSALLDSAEKLAPGKKVEENEQESKPAGSKRSEAGSIVDKGQVSSAPEECRSFMSGRPSQTPEHDEQMQGANLGRKDFWRKMFKSQSAASDTSSQSEQDTSECTTAHSGTTSDRRARSRSRRISLRKKLKLPIGKRNWLKRSSLSGLADGVEDLLDISSVDRLSFIRQSSKVKFTSAVKLSEGGPGSGMENGRDEEENFFKRLGCHSFDDHLSPNQDGGKSKNVVNLGAIRQGMKRFQFLLNCCEPGTIPDASILAAALDLEAPVVARAALFLECARFVHRCNRGNWPEWMKGHHVNITKKGLSRGRSPIVGNKRNQKLQWNAAKLFYQWGDAIGVRLNELCHGESESPANLLGLIYDEETKRRLRKEDEEEDFLDDSTVNPSKCGCPFALKMAACQLLLEITTFLRETFSCLPRPRTEPLVDLESCRLRLDPELDRHRYERKISFAGVLDENEDSKDSLHSSSHTLKSDAGVEEKKEGSPWSASEPSIEPEGMSNAGAEENYHRNMSWLHVMILLCNQQSFICTHVDYCHPHCYLHHSRSCARLVRAIKLLYGDSVDSLRESSNISSVALRGKKQKECSDKSCLRTPSLKKRVSDANLEGKKDSGMLKYIRLQVMSLSPAPLSLLIKAAPILTEEMYGDIQPAAWELLLSMDEHMAGAAAAMFLLCAVKVPEAVSDMLMSEFHHPETVQRLNAVLKFHTLWRFRYQVWPRMEEGAQQIFKIPPPSINFTLPSPVLGMPSVPMFDPPWVPQCSGSVQDPINEDQSKSFSARAVSRSHQRAEHILKNLQQEEEKKRLGREASLITAIPITQEACYEPTCTPNSEPEEEVEEVTNLASRRLSVSPSCTSSTSHRNYSFRRGSVWSVRSAVSAEDEEHTTEHTPNHHVPQPPQAVFPACICAAVLPIVHLMEDGEVREDGVAVSAVAQQVLWNCLIEDPSTVLRHFLEKLTISNRQDELMYMLRKLLLNIGDFPAQTSHILFNYLVGLIMYFVRTPCEWGMDAISATLTFLWEVVGYVEGLFFKDLKQTMKKEQCEVKLLVTASMPGTKTLVVHGQNECDIPTQLPVHEDTQFEALLKECLEFFNIPESQSTHYFLMDKRWNLIHYNKTYVRDIYPFRRSVSPQLNLVHMHPEKGQELIQKQVFTRKLEEVGRVLFLISLTQKIPTAHKQSHVSMLQEDLLRLPSFPRSAIDAEFSLFSDPQAGKELFGLDTLQKSLWIQLLEEMFLGMPSEFPWGDEIMLFLNVFNGALILHPEDSALLRQYAATVINTAVHFNHLFSLSGYQWILPTMLQVYSDYESNPQLRQAIEFACHQFYILHRKPFVLQLFASVAPLLEFPDAANNGPSKGVSAQCLFDLLQSLEGETTDILDILELVKAEKPLKSLDFCYGNEDLTFSISEAIKLCVTVVAYAPESFRSLQMLMVLEALVPCYLQKLKRQTSQVETVPAAREEIAATAALATSLQALLYSVEVLTRPMTAPQMSRCDQGHKGTTTANHTMSSGVNTRYQEQGAKLHFIRENLHLLEEGQGIPREELDERIAREEFRRPRESLLNICTEFYKHCGPRLKILQNLAGEPRVIALELLDVKSHMRLAEIAHSLLKLAPYDTQTMESRGLRRYIMEMLPITDWTAEAVRPALILILKRLDRMFNKIHKMPTLRRQVEWEPASNLIEGVCLTLQRQPIISFLPHLRSLINVCVNLVMGVVGPSSVADGLPLLHLSPYLSPPLPFSTAVVRLVALQIQALKEDFPLSHVISPFTNQERREGMLLNLLIPFVLTVGSGSKDSPWLEQPEVQLLLQTVINVLLPPRIISTSRSKNFMLESSPAHCSTPGDAGKDLRREGLAESTSQAAYLALKVILVCFERQLGSQWYWLSLQVKEMALRKVGGLALWDFLDFIVRTRIPIFVLLRPFIQCKLLAQPAENHEELSARQHIADQLERRFIPRPLCKSSLIAEFNSELKILKEAVHSGSAYQGKTSISTVGTSTSAYRLSLATMSRSNTGTGTVWEQDSEPSQQASQDTLSRTDEEDEENDSISMPSVVSEQEAYLLSAIGRRRFSSHVSSMSVPQAEVGMLPSQSEPNVLDDSQGLAAEGSLSRVASIQSEPGQQNLLVQQPLGRKRGLRQLRRPLLSRQKTQTEPRNRQGARLSTTRRSIQPKTKPSADQKRSVTFIEAQPEPAAAPTDALPATGQLQGCSPAPSRKPEAMDEPVLTSSPAIVVADLHSVSPKQSENFPTEEGEKEEDTEAQGATAHSPLSAQLSDPDDFTGLETSSLLQHGDTVLHISEENGMENPLLSSQFTFTPTELGKTDAVLDESHV

CLUSTAL format alignment by MAFFT (v7.511)

KAG1050648 M----SQEDQNDLVKNYKEESSRD------------------ATLDKQYKKKTKHF----

Homo_sapie MVKRKSSEGQ-------EQDGGRGIPLPIQTFLWRQTSAFLRPKLGKQYEASCVSFERVL

* *.*.* :::..*. ..*.***: . *

KAG1050648 -------------------SAW---------------------------DKLRENVSEY-

Homo_sapie VENKLHGLSPALSEAIQSISRWELVQAALPHVLHCTATLLSNRNKLGHQDKL--GVAETK

* * *** .*:*

KAG1050648 --------------------------------SSDTLYHATSDPSRPNMSKMSS------

Homo_sapie LLHTLHWMLLEAPQDCNNERFGGTDRGSSWGGSSSAFIHQVENQGSPGQPCQSSSNDEEE

**.:: * ..: . *. . **

KAG1050648 ---------------------------------------------PM--HKKQMYNPF--

Homo_sapie NNRRKIFQNSMATVELFVFLFAPLVHRIKESDLTFRLASGLVIWQPMWEHRQPGVSGFTA

** *:: . *

KAG1050648 ----LR-----------NQHTTTAENPLHDKINLTNTTLVAA--QAAGMS----------

Homo_sapie LVKPIRNIITAKRSSPINSQSRTCESPNQDARHLEGLQVVCETFQSDSISPKATISGCHR

:* *.:: *.*.* :* :* . :*. *: .:*

KAG1050648 --SFVRNFKSKVGAGR---HNGAI----------YAT--STAVQQDI-------------

Homo_sapie GNSFDGSLSSQTSQERGPSHSRASLVIPPCQRSRYATYFDVAVLRCLLQPHWSEEGTQWS

** .:.*:.. * *. * *** ..** : :

KAG1050648 --YRLERDLEKLLSHM---------------------------------NNRSQNASF--

Homo_sapie LMYYLQR-----LRHMLEEKPEKPPEPDIPLLPRPRSSSMVAAAPSLVNTHKTQDLTMKC

* *:* * ** .:::*: ::

KAG1050648 TESESSNHNTVYS---------------SDSLALN-------------------------

Homo_sapie NEEEKSLSSEAFSKVSLTNLRRSAVPDLSSDLGMNIFKKFKSRKEDRERKGSIPFHHTGK

.*.*.* . .:* *..*.:*

KAG1050648 ------------------------NFSVKSAS--------------KKTIF---------

Homo_sapie RRPRRMGVPFLLHEDHLDVSPTRSTFSFGSFSGLGEDRRGIEKGGWQTTILGKLTRRGSS

.**. * * :.**:

KAG1050648 ------------HRHSQAVL-----DPSTQHNEKHLDVTRISANITSIM-----------

Homo_sapie DAATEMESLSARHSHSHHTLVSDLPDPSNSHGEN--TVKEVRSQISTITVATFNTTLASF

* **: .* ***..*.*: *..: ::*::*

KAG1050648 -----DIVNKH--KSATRLPFTS---EILAVLSIPFDRKPQKTEDCIQALDAFDHIRSRF

Homo_sapie NVGYADFFNEHMRKLCNQVPIPEMPHEPLACANLP----RSLTDSCIN-----------Y

*:.*:* * ..::*:.. * ** .:* . *:.**: :

KAG1050648 TLLESSEHFEQVLFCCSILGSSGI----------------DLKEKILDILKKLVE-----

Homo_sapie SYLEDTEHIDGT---NNFVHKNGMLDLSVVLKAVYLVLNHDISSRICDVALNIVECLLQL

: **.:**:: . .:: ..*: *:..:* *: ::**

KAG1050648 ---PC------------------------FTYPNYLPSTPTAFHALAYALATS-------

Homo_sapie GVVPCVEKNRKKSENKENETLEKRPSEGAFQFKGVSGSSTCGFGGPAVSGAGDGGGEEGG

** * : . *:. .* . * : * .

KAG1050648 ---------------------FAKQTQNIELQEKK--TTYDLVRSGILSLLDNL------

Homo_sapie GGDGGGGGGDGGGGGGGGGGPYEKNDKNQEKDESTPVSNHRLALTMLIKIVKSLGCAYGC

: *: :* * :*.. :.: *. : ::.::..*

KAG1050648 ANGKLISVQGDQW--TTYFHTTQAASASLAKICMVESLCKCLMVSICRQTSFHERNAELF

Homo_sapie GEGHR-GLSGDRLRHQVFRENAQNCLTKLYKLDKMQF----------RQT----------

.:*: .:.**: .: ..:* . :.* *: :: ***

KAG1050648 LNNLIQKD--QDIPTLLELLQRYWTEPDTE------------------------ILPAYF

Homo_sapie MRDYVNKDSLNNVVDFLHALLGFCMEPVTDNKAGFGNNFTTVDNKSTAQNVEGIIVSAMF

:.: ::** ::: :*. * : ** *: *:.* *

KAG1050648 KSLGLLTELASEISLALSIEKLK-SSAIPLLFNFILEKSAPSNVYNYLNQAISGKIQKSV

Homo_sapie KS--LITRCASTTHELHSPENLGLYCDIRQLVQFI--KEAHGNVFRRV--ALSA------

** *:*. ** * *:* . * *.:** *.* .**:. : *:*.

KAG1050648 IFNIITMLLCFLSVDLLTAPSRSAPSSNTPRTPPNLTPQSSLNDMFISSPVLDDSGSEAP

Homo_sapie ---------------LLDSAEKLAPGKKVEENEQESKPAGS-------------KRSEAG

** :..: **..:. .. : .* .* . ***

KAG1050648 HII------VSPEE--AFLS----------QLLETAKAYLIEMWNYGYKSH--ICKCIEI

Homo_sapie SIVDKGQVSSAPEECRSFMSGRPSQTPEHDEQMQGANLGRKDFWRKMFKSQSAASDTSSQ

*: :*** :*:* : :: *: ::*. :**: .. .

KAG1050648 MLQDTSENQLAHTYQ-------------NLLFHIDIAIGEE--IAKVTIPILFKRILDTS

Homo_sapie SEQDTSECTTAHSGTTSDRRARSRSRRISLRKKLKLPIGKRNWLKRSSLSGLADGVEDLL

***** **: .* ::.:.**:. : : ::. * . : *

KAG1050648 PAPTPDFCSMLYQLSQ-RFRPL---------------------FYKPVVSCVA-------

Homo_sapie DISSVDRLSFIRQSSKVKFTSAVKLSEGGPGSGMENGRDEEENFFKR-LGCHSFDDHLSP

.: * *:: * *: :* . *:* :.* :

KAG1050648 SNDNDKVASLLKLMTSLRQYLSGVQFWMQ--------DAEMI-------------NVLLL

Homo_sapie NQDGGKSKNVVNL-GAIRQGMKRFQFLLNCCEPGTIPDASILAAALDLEAPVVARAALFL

.:*..* .:::* ::** :. .** :: **.:: .*:*

KAG1050648 S----------------------DVGPKKLQRETSSLQQNHTSSTNLLTWGSTTL-----

Homo_sapie ECARFVHRCNRGNWPEWMKGHHVNITKKGLSRGRSPIVGNKRNQK--LQWNAAKLFYQWG

. :: * *.* *.: *: ... * *.::.*

KAG1050648 ------------GQCIIATELVWVLKELREKQKDPDRNMEEDEVAKKFLIDLERRLALFL

Homo_sapie DAIGVRLNELCHGESESPANLLGLIYDEETKRRLRKEDEEED-----FLDD--------S

*:. .::*: :: : . *:: ..: *** ** *

KAG1050648 TAKEKKVLVPLPLRIILCNL------FADARFFCYTTHRPGWL----TCAIN--------

Homo_sapie TVNPSKCGCPFALKMAACQLLLEITTFLRETFSCLPRPRTEPLVDLESCRLRLDPELDRH

*.: .* *:.*:: *:* * * * . *. * :* :.

KAG1050648 -------------------------------------------W-ATQPRTNDIG-SQAI

Homo_sapie RYERKISFAGVLDENEDSKDSLHSSSHTLKSDAGVEEKKEGSPWSASEPSIEPEGMSNAG

* *::* : * *:*

KAG1050648 SETFLDENLS-----------ETGFCSN-DMSLPVLNANHIEEALIMFQRMQNTYLDVVE

Homo_sapie AEENYHRNMSWLHVMILLCNQQSFICTHVDYCHPHCYLHHSRSCARLVRAIKLLYGDSVD

:* ..*:* :: :*:: * . * :* ... :.: :: * * *:

KAG1050648 YFRDESSTSHGDSHGTRQAASSLLFSNTQMTENLKYGRRHLAETMYPINQSSSAALNLNP

Homo_sapie SLRESSNISSVALRGKKQKECS--DKSCLRTPSLK-------------KRVSDANL----

:*:.*. * :*.:* .* .. * .** :: *.* *

KAG1050648 PEFDHLENNEGESNSIMLDLAKHKFDGIEEINQDPFGSVFSLLAAVYSTLSSHELGRLLK

Homo_sapie ---------EGKKDSGML-----KYIRLQVMSLSP--APLSLLIKAAPILTEEMYGDIQP

**:.:* ** *: :: :. .* : :*** . . *:.. * :

KAG1050648 PLWKTYMD-DKKAASFTPAAFLLMECGEKIPKLTTEMF------SQALQRLNILNQSYIQ

Homo_sapie AAWELLLSMDEHMAGAAAAMFLL--CAVKVPEAVSDMLMSEFHHPETVQRLNAVLKFHTL

. *: :. *:: *. :.* *** *. *:*: .::*: .:::**** : : :

KAG1050648 ISSRNKPF-RGDGGA---FATP------FLPTD-LGSNEFSLDEPRWMSKLKNTSNLPIE

Homo_sapie WRFRYQVWPRMEEGAQQIFKIPPPSINFTLPSPVLGMPSVPMFDPPWVPQCSGSVQDPI-

* : : * : ** * * **: ** ...: :* *:.: ..: : **

KAG1050648 LKRQIQELGWNEDDN--------------AE------EQEAFKKAL--------------

Homo_sapie ----------NEDQSKSFSARAVSRSHQRAEHILKNLQQEEEKKRLGREASLITAIPITQ

***:. ** :** ** *

KAG1050648 --------TP------------------LGLLPSLFLKDDNEDTNDADNPNIVVIRERGK

Homo_sapie EACYEPTCTPNSEPEEEVEEVTNLASRRLSVSPSC--------TSSTSHRNYSF--RRGS

** *.: ** *..:.: * . .**.

KAG1050648 IVDI-SKIITRRKRTATVHS----------------FTAMFLTTVDLLMD-----DFAGV

Homo_sapie VWSVRSAVSAEDEEHTTEHTPNHHVPQPPQAVFPACICAAVLPIVHLMEDGEVREDGVAV

: .: * : :. :. :* *: : * .*. *.*: * * ..*

KAG1050648 NNVLRELIEQFLKDDPALFLRAFLNNLG-KHKADSLKDTLTRIRYIISMQRKL-------

Homo_sapie SAVAQQVLWNCLIEDPSTVLRHFLEKLTISNRQDEL----------MYMLRKLLLNIGDF

. * :::: : * :**: .** **::* .:: *.* : * ***

KAG1050648 PPGLTHILFNYLAGMLKWIVRENQPDGLKFMTLVHPILAELTLSTNDLSVRDL----RKN

Homo_sapie PAQTSHILFNYLVGLIMYFVRTPCEWGMDAISATLTFLWEVVGYVEGLFFKDLKQTMKKE

*. :*******.*:: ::** *:. :: . .:* *:. .:.* .:** :*:

KAG1050648 KIE-HLLVSTG----KFWFTHEQPADMFPRYLS-NEQHSFAVL--------NIPQQI---

Homo_sapie QCEVKLLVTASMPGTKTLVVHGQNECDIPTQLPVHEDTQFEALLKECLEFFNIPESQSTH

: * :***::. * ..* * :* *. :*: .* .* ***:.

KAG1050648 FFVACLRISHIQFLINYLARFPREVYAVKKTLQDYEPM-------PVPGSKWETRQLMEK

Homo_sapie YFLMDKRWN----LIHYNKTYVRDIYPFRRSVS---PQLNLVHMHPEKG-----QELIQK

:*: * . **:* : *::*..::::. * * * ::*::*

KAG1050648 AYYPD-----------LSRRKNRQSNHKQ------------------EPLDTE-----DS

Homo_sapie QVFTRKLEEVGRVLFLISLTQKIPTAHKQSHVSMLQEDLLRLPSFPRSAIDAEFSLFSDP

:. :* :: : *** ..:*:* *.

KAG1050648 QQHFDKEMLSL--MRARIWLRFIDVLLNGLNKNYNDHDELERILQGVNTIMIEHSHDFGI

Homo_sapie QA--GKELFGLDTLQKSLWIQLLEEMFLGMPSEFPWGDEIMLFLNVFNGALILHPEDSAL

* .**::.* :: :*::::: :: *: .:: **: :*: .* :* *..* .:

KAG1050648 IGQILILYTRMVTKFKRLFKSNRGYIIFLPALFKVFCESEDNPQITSAITFAWCRFYAVH

Homo_sapie LRQYAATVINTAVHFNHLF-SLSGYQWILPTMLQVYSDYESNPQLRQAIEFACHQFYILH

: * . ..:*::** * ** :**::::*:.: *.***: .** ** :** :*

KAG1050648 EEAFVFQMLGALVPNILMIYGQSDEVGHWMIENLFKLMQAMDNPPHLGSAPDTLGLQLQV

Homo_sapie RKPFVLQLFASVAP-------------------LLEFPDAANNGPSKGVSAQCLFDLLQ-

.:.**:*::.::.* *::: :* :* * * :.: * **

KAG1050648 ELDDHERSIQDRIDTASSQVSSALSVSIFKPLARGVTAPISQMEISTYNNR--PFKLEDF

Homo_sapie SLEGETTDILDILELVKAE------------------KPLKSLDF-CYGNEDLTFSISEA

.*:.. .* * :: ..:: *:..::: *.*. .*.:.:

KAG1050648 IKLFLTVIAYDPGSLRAEQFVKVFTFLIPFFLEQSHLKSMMHE----------GASALVN

Homo_sapie IKLCVTVVAYAPESFRSLQMLMVLEALVPCYLQKLKRQTSQVETVPAAREEIAATAALAT

*** :**:** * *:*: *:: *: *:* :*:: : :: * .::**..

KAG1050648 VFVKFSKSVKSV------------------VTTNSHTTSGEYQGISSSDSRLPGSNNAAN

Homo_sapie SLQALLYSVEVLTRPMTAPQMSRCDQGHKGTTTANHTMS-----------------SGVN

: : **: : .** .** * ...*

KAG1050648 TGPKAESA------------QNAYGKQWQQNDRFTIKQEF-------ITLVHKYMKHGGT

Homo_sapie TRYQEQGAKLHFIRENLHLLEEGQGIPREELDERIAREEFRRPRESLLNICTEFYKHCGP

* : :.* ::. * :: *. ::** :.: :: ** *.

KAG1050648 -------------------LSEVSHEKMAQIIRIVLR----DYRNIKGLTVK--------

Homo_sapie RLKILQNLAGEPRVIALELLDVKSHMRLAEIAHSLLKLAPYDTQTMESRGLRRYIMEMLP

*. ** ::*:* : :*: * :.::. ::

KAG1050648 -TDWIKDYLVVSMYSMVDTRNYTKSLKKVLSEIYA----QYRVQWKTVDASDLYEGLAIM

Homo_sapie ITDWTAEAVRPALILIL------KRLDRMFNKIHKMPTLRRQVEWEP--ASNLIEGVCLT

*** : : :: :: * *.:::.:*: : :*:*:. **:* **:.:

KAG1050648 LEQGQGKAIM----------MNDLAGLVKEKFVSFGLTMATLTDWQNNTKGQVKFCNSLV

Homo_sapie LQRQPIISFLPHLRSLINVCVNLVMGVVGPSSVADGLPLLHLSPYLSPP---LPFSTAVV

*:: ::: :* : *:* . *: **.: *: : . . : *..::*

KAG1050648 RLIIAILENSSQD-----VFAEIERQVPSVGLIGRVVIPLCLQYNLQWEYSTVGISSTSE

Homo_sapie RLVALQIQALKEDFPLSHVISPFTNQERREGMLLNLLIPFVL---------TVGSGSKD-

**: :: .:* *:: : .* *:: .::**: * *** .*..

KAG1050648 LNSANNWTR------LLNFIAGVCSHTSLLK-SKSSGFSLSQFTTAMGQTNIEDEVDMSD

Homo_sapie ----SPWLEQPEVQLLLQTVINVLLPPRIISTSRSKNFMLES-SPAHCSTPGDAGKDLR-

. * . **: : .* . ::. *:*..* *.. :.* .* : *:

KAG1050648 FPEAKDGKQTPQSVAVLFSLSLIAIKIILIRSHNTFNDLKGS-WSQIAFFIRNTLVFGQT

Homo_sapie ----REGLAESTSQA-----AYLALKVILV----CFERQLGSQWYWLSL---------QV

::* . * * : :*:*:**: *: ** * ::: *.

KAG1050648 LKLLKAKVGRSDSYNLASPSDVLSPGMPSPSFFSPLGLSSPGASPYLSSNNPSLSPDLRA

Homo_sapie KEMALRKVG---------------------------GLA---------------------

:: *** **:

KAG1050648 NFSSNAPLGTGTIYDFTTWRFLEFVVCFRTPLFVLLKDFI-----------HEKL-----

Homo_sapie -----------------LWDFLDFIVRTRIPIFVLLRPFIQCKLLAQPAENHEELSARQH

* **:*:* * *:****: ** **:*

KAG1050648 ----------------------------------------------GQMGPNVGSYRNSL

Homo_sapie IADQLERRFIPRPLCKSSLIAEFNSELKILKEAVHSGSAYQGKTSISTVGTSTSAYRLSL

. :*....:** **

KAG1050648 YS---------------------------PRASFNFPQNENTRWKSWGAEHSSVNTSGDG

Homo_sapie ATMSRSNTGTGTVWEQDSEPSQQASQDTLSRTDEEDEENDSISMPSVVSEQEAYLLSAIG

: .*:. : :*:. * :*:.: *. *

KAG1050648 QSNANASAHSIVIPKLNIEDTGGVDAETLPTFGLGLHKSNSSASPGHVSHSPTGYSETDS

Homo_sapie RRRFSSHVSSMSVPQAEV---GMLPSQSEPNV---LDDSQGLAAEGSLSRVASIQSEPGQ

: . .: . *: :*: :: * : ::: *.. *..*:. *: * :*: .: **...

KAG1050648 S----------------------SPRSPTTNPFYKQQQHMS-----------PGADVSKE

Homo_sapie QNLLVQQPLGRKRGLRQLRRPLLSRQKTQTEPRNRQGARLSTTRRSIQPKTKPSADQKRS

. * :.. *:* :* ::* *.** .:.

KAG1050648 NNLHLLQAETITSVVNVQVAVGF----------------KPAL---PWITMD-----SRD

Homo_sapie VTFIEAQPEPAAAPTDALPATGQLQGCSPAPSRKPEAMDEPVLTSSPAIVVADLHSVSPK

.: *.*. :: .:. *.* :*.* * *.: * .

KAG1050648 RSKPWNKKEAVARIANEWQ-------LLLQLST-------ET-------DPLVNKANNNG

Homo_sapie QSENFPTEEGEKEEDTEAQGATAHSPLSAQLSDPDDFTGLETSSLLQHGDTVLHISEENG

:*: : .:*. . .* * * *** ** *.::: :::**

KAG1050648 S-----SANISASPL-------------V

Homo_sapie MENPLLSSQFTFTPTELGKTDAVLDESHV

*:::: :* *

**UNC79**

>XP_012053306.1 hypothetical protein CNAG_06362 [Cryptococcus neoformans var. grubii H99]

MLSVPEGNTNDDARKALGIRPGGQPPPQLKLDHTNFEDIPRSPALDYPPSPRSPTSSKLPSSPIPPSPTSPEPSRTFRRATRVGRHVLSTKKIQKISSSRGLYETQKLLLYLLDTLEQRETAPDILDRAAISAREISGRLKGKGKGKVLRLGHAIAAAASSSSSIGSSRTHHAMTSTTGVGSEDYEDHIVLDEGDWDTEASYNLVEQTRGLFVLAEKQQLDLFADTGDNDVPILDATPVKSKRRAGRFSSVSPSVAFTKGSSQAAPSSPDISFASTLSPSRTSTSTIPSIPGPYLLERTLNVLQSLLSVDCLHRTRTFRPLCPPNALQAACLDIAAYLYQKGEVGTKVRVVGMVIDGFYGMGEGMEERICEWLEGRMGDLLGRLARERGDFKKPKSDDVEWIDPFSKQDSNTSRNAALPTFAISSESSDSVLRKVSGTQGWMRFSPTSPSFSMFHHGILGLLSIQSLRDASSTAVEIAALVPRILMAITSTIDLTQSKLTTIYRVHRLLSLVLNAKPDSSLDLLTIIAHAPPLSRRTAAEILATFYPNAAGHNTVARRLPSSSYVAQRTKWETGQFGVLGEDETEEHHFVPWRVSSHDDPTAEVVRCEVCESEIHGFGIKCTMCKGQRHLRCYDGSGKTRKGVYQYDVVTLSPTSTSSQLSHAKFCPSLPRLEEVLLSPPHGSECTHRKAGQHRLDLIHLFTLTLCDECHFPLWGVSKQGYTCQNGCQRFFHSHCLEKMEETGVGECRYGREVVVDEISEAGNNPFVITRDKLQSSFHQHYNGFLPSKDELKGKTFDEIGVIFGTIWIQYQLIQNGLSSGSLRVSNEDKKATSDPLGLKPILKTYEELLQSHEQAISTAAADFTHAATLEKPLGTGYLFSEKFLGYCTALIRAPTTSSSTLSREGEHLGDGFLTPQGLSPSLEEGDKVDRCYETLPLGIIVQSLATDLSLSNKVAATAFLNHLSLVGFISIFDTASLTSQYLAKRREIKVSFTLPLLMDSSPNTELLVLAIEALLDDLDLTMNEQGLRLLVTRAWPSLLCAPYALERLGKACVSWVISEEDCLRDIIKKYASKHRRIPGVRPAAGAQKGSVDMYKQDRQRLRRMFVEPWLKALHDQDPALYVHIVYEQCKLVAANVAMEDLSGASEEQVASRMAGVAVSKMTSMGEAGMLFSTVMELLTAWLEDLGPLADHDVAYRALPRLLNHQPADSADIFGLSLSTAQAGPADLARVCRWMRVLSYSGVEIPWELLLSLVDLQAGSPAFTFDGSVGGASAEAKLDLVIAINSNRAVIEPQTFASACSRLAVGVFCDMGKHEKMEPLELELVKRTMLLILQAYGVPVDEVAETTLGVGMPSTGQPPTITKKRQSPIVNVRFPLNADMVVGAATLLERTSCPDEMVLDFLWLLSTKAGMVDDPVGFLHHTCSKLYEMIWPLIGLPIDRRSRARVLLKLLSVNSAPLERIIHAQLEFSPEARAQARERLLIFILELADTSVNYELSNWRAAMVGLVLLFFDVLLDPRDVTPDNIIILKTLQPTQLNAMSMCFEEHLVKSSDERRLVLLSRLSRLRMNVPQWAIISWTTIDELLAEEVASLTQFKRADQSQTDSGRVDSQNVAYSLIFLGLEMLAAGVPITWIAAQRFQQRVAAACALPWFNPPVFTAVVLPGLRSVLDSPIRIMISGETFESKVKKTVLVGSLFVPVVIDFAQELKKYDVAVQRILLDILMVTFFKQDVTRVELSTYSAVQKVADFVLTGECSENRLLALQILQIAVTKVERDKIIRAVPPAFNTVAKVLVKELEAEYGDPAVVEQSQLFLRNIVKGFGRSGLYLQLFRNESESYDLSSPHETSLAKALQILHNEQEREALPQASLFDNVFHDLLDVTKRPRQQVIHIIEAFARFATSFEGHLSEEAAQDFGTFVNRLSKLIAEWSFDFDPNPILQSCAKILDRTPSASLVPLLSQVSTILNHCMGHFAVKRATAIRLLESGETASRKAQTENQIRIVLFEMAGAAINGLPVTPEGLYTLLKYLTSDAFSRPAPDSRVSSEQIRVLSDASPGCVHILLNGHPALESNLMSAEVLVAVLNEAGTVLCQTEMVVPGAISRNVTQLTLDAASSQVNFFLYLLLSSLDLTMGPARSRLLSLYPLLSRATSLCLRAASDYMSLQDVEGHGTFLISLAFMVMRLALLAVRDGPVSNEDREKGAGDEDAVDILWTRIWPEWIRLFRLSFDPNCVNGALRTVTHSVFLDLLTFLGGIYSPILLRHTESLSVNLAMLVQYQETLGVAPSGKLQKATQIMEKVGTHSVGGVADRMGMMESLKADLLAMERMKILNK

>Homo_sapiens_NP_001333147.1

MSTKAEQFASKIRYLQEYHNRVLHNIYPVPSGTDIANTLKYFSQTLLSILSRTGKKENQDASNLTVPMTMCLFPVPFPLTPSLRPQVSSINPTVTRSLLYSVLRDAPSERGPQSRDAQLSDYPSLDYQGLYVTLVTLLDLVPLLQHGQHDLGQSIFYTTTCLLPFLNDDILSTLPYTMISTLATFPPFLHKDIIEYLSTSFLPMAILGSSRREGVPAHVNLSASSMLMIAMQYTSNPVYHCQLLECLMKYKQEVWKDLLYVIAYGPSQVKPPAVQMLFHYWPNLKPPGAISEYRGLQYTAWNPIHCQHIECHNAINKPAVKMCIDPSLSVALGDKPPPLYLCEECSERIAGDHSEWLIDVLLPQAEISAICQKKNCSSHVRRAVVTCFSAGCCGRHGNRPVRYCKRCHSNHHSNEVGAAAETHLYQTSPPPINTRECGAEELVCAVEAVISLLKEAEFHAEQREHELNRRRQLGLSSSHHSLDNADFDNKDDDKHDQRLLSQFGIWFLVSLCTPSENTPTESLARLVAMVFQWFHSTAYMMDDEVGSLVEKLKPQFVTKWLKTVCDVRFDVMVMCLLPKPMEFARVGGYWDKSCSTVTQLKEGLNRILCLIPYNVINQSVWECIMPEWLEAIRTEVPDNQLKEFREVLSKMFDIELCPLPFSMEEMFGFISCRFTGYPSSVQEQALLWLHVLSELDIMVPLQLLISMFSDGVNSVKELANQRKSRVSELAGNLASRRVSVASDPGRRVQHNMLSPFHSPFQSPFRSPLRSPFRSPFKNFGHPGGRTIDFDCEDDEMNLNCFILMFDLLLKQMELQDDGITMGLEHSLSKDIISIINNVFQAPWGGSHTCQKDEKAIECNLCQSSILCYQLACELLERLAPKEESRLVEPTDSLEDSLLSSRPEFIIGPEGEEEENPASKHGENPGNCTEPVEHAAVKNDTERKFCYQQLPVTLRLIYTIFQEMAKFEEPDILFNMLNCLKILCLHGECLYIARKDHPQFLAYIQDHMLIASLWRVVKSEFSQLSSLAVPLLLHALSLPHGADIFWTIINGNFNSKDWKMRFEAVEKVAVICRFLDIHSVTKNHLLKYSLAHAFCCFLTAVEDVNPAVATRAGLLLDTIKRPALQGLCLCLDFQFDTVVKDRPTILSKLLLLHFLKQDIPALSWEFFVNRFETLSLEAQLHLDCNKEFPFPTTITAVRTNVANLSDAALWKIKRARFARNRQKSVRSLRDSVKGPVESKRALSLPETLTSKIRQQSPENDNTIKDLLPEDAGIDHQTVHQLITVLMKFMAKDESSAESDISSAKAFNTVKRHLYVLLGYDQQEGCFMIAPQKMRLSTCFNAFIAGIAQVMDYNINLGKHLLPLVVQVLKYCSCPQLRHYFQQPPRCSLWSLKPHIRQMWLKALLVILYKYPYRDCDISKILLHLIHITVNTLNAQYHSCKPHATAGPLYSDNSNISRYSEKEKEEDSVFDESDIHDTPTGPCNKESQTFFARLKRIGGSKMVKYQPVEMNVQRSEIELAEYRETGALQDSLLHCVREESIPKKKLRSFKQKSLDIGNADSLLFTLDEHRRKSCIDRCDIEKPPTQAAYIAQRPNDPGRSRQNSATRPDNSEIPENPAMEGFPDARRPVIPEVRLNCMETFEVKVDSPVKPAPKEDLDLIDLSSDSTSGPEKHSILSTSDSDSLVFEPLPPLRIVESDEEEETMNQGDDGPSGKNAASSPSVPSHPSVLSLSTAPLVQVSVEDCSKDFSSKDSGNNQSAGNTDSALITLEDPMDAEGSSKPEELPEFSCGSPLTLKQKRDLLQKSFALPEMSLDDHPDPGTEGEKPGELMPSSGAKTVLLKVPEDAENPTESEKPDTSAESDTEQNPERKVEEDGAEESEFKIQIVPRQRKQRKIAVSAIQREYLDISFNILDKLGEQKDPDPSTKGLSTLEMPRESSSAPTLDAGVPETSSHSSISTQYRQMKRGSLGVLTMSQLMKRQLEHQSSAPHNISNWDTEQIQPGKRQCNVPTCLNPDLEGQPLRMRGATKSSLLSAPSIVSMFVPAPEEFTDEQPTVMTDKCHDCGAILEEYDEETLGLAIVVLSTFIHLSPDLAAPLLLDIMQSVGRLASSTTFSNQAESMMVPGNAAGVAKQFLRCIFHQLAPNGIFPQLFQSTIKDGTFLRTLASSLMDFNELSSIAALSQLLEGLNNKKNLPAGGAMIRCLENIATFMEALPMDSPSSLWTTISNQFQTFFAKLPCVLPLKCSLDSSLRIMICLLKIPSTNATRSLLEPFSKLLSFVIQNAVFTLAYLVELCGLCYRAFTKERDKFYLSRSVVLELLQALKLKSPLPDTNLLLLVQFICADAGTKLAESTILSKQMIASVPGCGTAAMECVRQYINEVLDFMADMHTLTKLKSHMKTCSQPLHEDTFGGHLKVGLAQIAAMDISRGNHRDNKAVIRYLPWLYHPPSAMQQGPKEFIECVSHIRLLSWLLLGSLTHNAVCPNASSPCLPIPLDAGSHVADHLIVILIGFPEQSKTSVLHMCSLFHAFIFAQLWTVYCEQSAVATNLQNQNEFSFTAILTALEFWSRVTPSILQLMAHNKVMVEMVCLHVISLMEALQECNSTIFVKLIPMWLPMIQSNIKHLSAGLQLRLQAIQNHVNHHSLRTLPGSGQSSAGLAALRKWLQCTQFKMAQVEIQSSEAASQFYPL

CLUSTAL format alignment by MAFFT (v7.511)

XP_0120533 MLS-------------------------VPEG----NT----------------------

Homo_sapie MSTKAEQFASKIRYLQEYHNRVLHNIYPVPSGTDIANTLKYFSQTLLSILSRTGKKENQD

* : **.* **

XP_0120533 ------------------------------------------------------------

Homo_sapie ASNLTVPMTMCLFPVPFPLTPSLRPQVSSINPTVTRSLLYSVLRDAPSERGPQSRDAQLS

XP_0120533 ----------------------------------------------NDD-----------

Homo_sapie DYPSLDYQGLYVTLVTLLDLVPLLQHGQHDLGQSIFYTTTCLLPFLNDDILSTLPYTMIS

***

XP_0120533 -----------------------------ARK----------------------------

Homo_sapie TLATFPPFLHKDIIEYLSTSFLPMAILGSSRREGVPAHVNLSASSMLMIAMQYTSNPVYH

:*:

XP_0120533 ------------------------------------------------------------

Homo_sapie CQLLECLMKYKQEVWKDLLYVIAYGPSQVKPPAVQMLFHYWPNLKPPGAISEYRGLQYTA

XP_0120533 --------------------------ALGIRPGGQPPP---------QLKLDHTNFEDIP

Homo_sapie WNPIHCQHIECHNAINKPAVKMCIDPSLSVALGDKPPPLYLCEECSERIAGDHSEWLIDV

:*.: *.:*** :: **:::

XP_0120533 RSPALDYPPSPRSPTSSKLPSSPIPPSPTSPEPSRTFRRATRVGRHVLSTKKIQKI--SS

Homo_sapie LLPQAEISAICQKKNCSSHVRRAVVTCFSAGCCGRHGNRPVRYCKRCHSNHHSNEVGAAA

* : .. :. ..*. .: .. :: .* .*..* :: *.:: ::: ::

XP_0120533 SRGLYETQKLLLYLLDTLEQRETAPDILDRAAISAREISGRLKGKGKGKVLRLGHAIAAA

Homo_sapie ETHLYQTSP------PPINTRECGAEEL----VCAVEAVISLLKEAEFHAEQREHEL---

. **:*. .:: ** ..: * :.* * * :.: :. : * :

XP_0120533 ASSSSSIGSSRTHHAMTSTTGVGSED--YEDHIVLDEGDW--------------------

Homo_sapie -NRRRQLGLSSSHHSLDNADFDNKDDDKHDQRLLSQFGIWFLVSLCTPSENTPTESLARL

. .:* * :**:: .: ..:* :::::: : * *

XP_0120533 ---------------DTEASYNLVEQTRGLFVLAEKQQLDLFADTG-DNDVPILDATPVK

Homo_sapie VAMVFQWFHSTAYMMDDEVG-SLVEKLKPQFV---TKWLKTVCDVRFDVMVMCLLPKPME

* *.. .***: : ** .: *. ..*. * * * ..*::

XP_0120533 SKRRAGRFSSVSPSVAFTKGSSQAAPSSPDISFASTLSPSRTSTSTIPSIPGPYLLERTL

Homo_sapie FARVGGYWD---------KSCSTVTQLKEGLNRILCLIPYNVINQSVWECIMPEWLEAIR

* .* :. *..* .: . .:. * * .. ..:: . * **

XP_0120533 NVLQSLLSVDCLHRTRTFR---------PLCP-------------------PNALQAACL

Homo_sapie TEVPD-------NQLKEFREVLSKMFDIELCPLPFSMEEMFGFISCRFTGYPSSVQEQAL

. : . :: : ** *** *.::* .*

XP_0120533 DIAAYLYQKGEVGTKVRVVGMVIDGFYGMGEGMEERICEWLEGRMGDLLGRLARERGDFK

Homo_sapie LWLHVLSELDIMVPLQLLISMFSDGVNSVKELANQR-----KSRVSELAGNLASRR----

* : . : . ::.*. **. .: * ::* :.*:.:* *.** .*

XP_0120533 KPKSDDVEWIDPFSKQDSNTSRNAALPTFAISSESSDSVLRKVSGTQGWMRFSPTSPSFS

Homo_sapie ---------VSVASDPGRRVQHNMLSPFHSPFQSPFRSPLR-----------SPFRSPFK

:. *. . ...:* * .: ... * ** ** ..*.

XP_0120533 MFHHGILGLLSIQSLRDASSTAVEIAALVPRILM--------AITSTIDLTQSKLTTIYR

Homo_sapie NFGHP--GGRTIDFDCEDDEMNLNCFILMFDLLLKQMELQDDGITMGLEHSLSK-DIISI

* * * :*: : .. :: *: :*: .** :: : ** *

XP_0120533 VHRLLSLVLNAKPDSSLDLLTI---IAHAPPLSRRTAAEILATFYPNAAGHNTVARRLPS

Homo_sapie INNVFQAPWGGSHTCQKDEKAIECNLCQSSILCYQLACELLERLAPKEESRLVEPTDSLE

::.::. ... .. * :* :.::. *. : *.*:* : *: .: . . .

XP_0120533 SSYVAQRTKWETGQFGVLGEDETEEHHFVPWRVSSHDDPTAEVVRCEVCESEIHGFGIKC

Homo_sapie DSLLSSRPEFIIGPEGEEEENPASKHGENPGN----------------CTEPVEHAAVK-

.* ::.*.:: * * *: :.:* * . * . :. .:*

XP_0120533 TMCKGQRHLRCYDGSGKTRKGVYQYDVVTLSPTSTSSQLSHAKFCPS---LPRLEEVLLS

Homo_sapie --------------NDTERKFCYQQLPVTLRLIYTIFQ-EMAKFEEPDILFNMLNCLKIL

... ** ** *** * * . *** . : *: : :

XP_0120533 PPHGSECTHRKAGQHRLDLIHLFTLTLCDECHFPLWGVSKQGYTCQNGCQRFFHSHCLEK

Homo_sapie CLHG-ECLYIARKDHPQFLAYIQDHMLIAS----LWRVVKSEFSQLSSLAVPLLLHALSL

** ** : :* * :: * . ** * *. :: .. : *.*.

XP_0120533 MEETGV-------------GECRYGREVVVDEISEAGNNPFVITRDKLQSSFHQHYNGFL

Homo_sapie PHGADIFWTIINGNFNSKDWKMRFEAVEKVAVICRFLDIHSVTKNHLLKYSLAHAFCCFL

. :.: : *: * *.. : * ... *: *: : : **

XP_0120533 PSKDELKGKTFDEIGVIFGTIWIQYQLIQNGLSSGSLRVSNEDKKATSDPLGLKPILKTY

Homo_sapie TAVEDVNPAVATRAGLLLDTI------KRPALQGLCLCLDFQFDTVVKD----RPTILSK

.: :::: . . *:::.** : .*.. .* :. : .....* :* : :

XP_0120533 EELLQSHEQAISTAAADF----THAATLEKPLGTGYLFSEKFLGYCTALIRAPTTSSSTL

Homo_sapie LLLLHFLKQDIPALSWEFFVNRFETLSLEAQL---HLDCNKEFPFPTT-ITAVRTNVANL

**: :* *.: : :* .: :** * :* .:* : : *: * * *. :.*

XP_0120533 S-------REGEHLGDGFLTPQGLSPSLE---EGDKVDRCYETLPLGIIVQSLATDLSLS

Homo_sapie SDAALWKIKRARFARNRQKSVRSLRDSVKGPVESKRALSLPETLTSKIRQQSPENDNTIK

* :.... : : :.* *:: *..:. ***. * ** .* ::.

XP_0120533 NKVAATAFLNHLSLVGFISIF------DTASLTSQYLAKRREIKVSFTLPLLMDSSPNTE

Homo_sapie DLLPEDAGIDHQTVHQLITVLMKFMAKDESSAESDISSAKAFNTVKRHLYVLLGYDQQEG

: :. * ::* :: :*::: * :* *: : : .*. * :*:. . :

XP_0120533 LLVLA-------------IEALLDDLDLTMNEQGLRLLVTRAWPSLLCAPYALERLGKAC

Homo_sapie CFMIAPQKMRLSTCFNAFIAGIAQVMDYNIN------------------------LGKHL

:::* * .: : :* .:* ***

XP_0120533 VSWVISEEDCLRDIIKKYASKHRRIPGVRPAAGAQKGSVDMYKQDRQRLRRMFVEP----

Homo_sapie LPLVVQ--------VLKYCS----CPQLR-----------HYFQQPPRCSLWSLKPHIRQ

:. *:. : **.* * :* * *: * ::*

XP_0120533 -WLKAL---------HDQD-PALYVHIV----------YEQCK-------LVAANVAMED

Homo_sapie MWLKALLVILYKYPYRDCDISKILLHLIHITVNTLNAQYHSCKPHATAGPLYSDNSNISR

***** :* * . : :*:: *..** * : * :.

XP_0120533 LSGASEEQVA-----------------------SRMAGVAVSKMTSMGEAGMLFSTVMEL

Homo_sapie YSEKEKEEDSVFDESDIHDTPTGPCNKESQTFFARLKRIGGSKMVKYQPVEMNVQR-SEI

* .:*: : :*: :. ***.. . * .. *:

XP_0120533 LTAWLEDLGPLAD---HDVAYRALPR----------------------LLNHQPADSADI

Homo_sapie ELAEYRETGALQDSLLHCVREESIPKKKLRSFKQKSLDIGNADSLLFTLDEHRRKSCIDR

* .: *.* * * * .::*: * :*: .. *

XP_0120533 FGLSLSTAQAG-----PADLARVCRWMRVLSYSGVEIPWELLLSLVDLQAGSPAF-----

Homo_sapie CDIEKPPTQAAYIAQRPNDPGR-SRQNSATRPDNSEIPENPAMEGFP-DARRPVIPEVRL

.:. ..:**. * * .* .* . .. *** : :. . :* *.:

XP_0120533 ----TFDGSVGGASAEA---KLDLVIAINSNRAVIEPQTFASACSRLAVGVFCDMGKHEK

Homo_sapie NCMETFEVKVDSPVKPAPKEDLDLIDLSSDSTSGPEKHSILSTSDS-------DSLVFEP

**: .*... * .***: ... : * ::: *:.. * .*

XP_0120533 MEPLELELVKRTMLLILQAYGVPVDEVAETTLGVGMPSTGQPPTITKKRQSPIVNVRFP-

Homo_sapie LPPLRIVESDEEEETMNQGDDGPSGKNAASS-----PSVPSHPSVLSLSTAPLVQVSVED

: **.: .. : *. . * .: * :: **. . *:: . :*:*:* .

XP_0120533 LNADMVVGAATLLERTSCPDEMVLDFLWLLSTKAGMVDDPVGFLHHTCSKLYEMIWPLIG

Homo_sapie CSKDFSSKDSGNNQSAGNTDSALI-----------TLEDPMD--AEGSSKPEELPEFSCG

. *: : : :. .*. :: ::**:. . .** *: *

XP_0120533 LPIDRRSRARVLLKLLSVNSAPLERIIHAQLEFSPEAR---AQARERLLIFILELADTSV

Homo_sapie SPLTLKQKRDLLQKSFALPEMSLDDHPDPGTEGEKPGELMPSSGAKTVLLKVPEDAENPT

*: :.: :* * ::: . .*: .. * . .. :.. : :*: : * *:...

XP_0120533 NYELSNWRAAMVGLVLLFFDVLLDP-RDVTPDNIIILKTLQPTQLNAMSMCFEEHLVKSS

Homo_sapie ESEKPDTSAES--------DTEQNPERKVEED-------------GAEESEFKIQIVPRQ

: * .: * *. :* *.* * .* . *: ::* .

XP_0120533 DERRLVLLSRLSRLRMNVPQWAIISWTTIDELLAEE--------VASLTQFKRADQSQTD

Homo_sapie RKQRKIAVSAIQR------EYLDISFNILDKLGEQKDPDPSTKGLSTLEMPRESSSAPTL

::* : :* :.* :: **:. :*:* :: :::* :.:..: *

XP_0120533 SGRVDSQNVAYSLIFLGLEMLAAGVPITWIAAQRFQQRVAAACALPWFNPPVFTAVVLPG

Homo_sapie DAGV-PETSSHSSISTQYRQMKRGSLGVLTMSQLMKRQLEHQSSAPHNISNWDTEQIQPG

.. * .:. ::* * . : * . :* ::::: .: * . * : **

XP_0120533 LRS----------VLDSPIRIMISGETFESKVKKTVLVGSLFVPVVIDFAQELKKYDVAV

Homo_sapie KRQCNVPTCLNPDLEGQPLR--MRGATKSSLLSAPSIV-SMFVPAPEEFTDEQPT-----

*. : ..*:* : * * .* :. . :* *:***. :*::* .

XP_0120533 QRILLDILMVTFFKQDVTRVELSTYSAVQKVADFVLTGECSENRLLALQILQIAVTKVER

Homo_sapie --------VMTDKCHDCGAILEEYDEETLGLAIVVLSTFIHLSPDLAAPLLLDIMQSVGR

::* :* : . . . :* .**: . ** :* : .* *

XP_0120533 DKIIRAVPPAFNTVAKVLVKELEAEYGDPAVVEQSQLFLRNIVKGFGRSGLYLQLFRNES

Homo_sapie ----LASSTTFSNQAESMMVP-----GNAAGVAKQ--FLRCIFHQLAPNGIFPQLFQSTI

* ..:*.. *: :: *:.* * :. *** *.: :. .*:: ***:.

XP_0120533 E---------SYDLSSPHETSLAKALQILHNEQEREALPQASLFDNVFHDLLDVTKRPRQ

Homo_sapie KDGTFLRTLASSLMDFNELSSIAALSQLLEGLNNKKNLPAGGA-----------------

: * :. . :*:* *:*.. :::: ** ..

XP_0120533 QVIHIIEAFARFATSFEGHLSEEAAQDFGTFVNRLSKLIAEWSFDFDPNPILQSCAKI--

Homo_sapie ----MIRCLENIATFMEALPMDSPSSLWTTISNQFQTFFAKLPCVLPLKCSLDSSLRIMI

:*..: .:** :*. :..:. : *: *::..::*: . : : *:*. :*

XP_0120533 -LDRTPSASLV-PLLSQVSTILNHCMGHFAVKRATAIRLLESGETASRKAQTENQI-RIV

Homo_sapie CLLKIPSTNATRSLLEPFSKLLSFVIQNAVFTLAYLVELCGLCYRAFTKERDKFYLSRSV

* : **:. . .**. .*.:*.. : : ... * :.* * * : : : * *

XP_0120533 LFEMAGAAINGLPVTPEGLYTLLKYLTSDAFSRPAPDSRVSSEQIRVL---SDASPGCVH

Homo_sapie VLELLQALKLKSPLPDTNLLLLVQFICADAGTKLAESTILSKQMIASVPGCGTAAMECVR

::*: * *:. .* *:::: :** :: * .: :*.: * : . *: **:

XP_0120533 ILLNGHPALESNLMSAEVLVAVLNEAGTVLCQTEMVVPGAISRNVTQLTLDAAS------

Homo_sapie QYINEVLDFMADMHTLTKLKSHMKTCSQPL--HEDTFGGHLKVGLAQIAAMDISRGNHRD

:* : ::: : * : :: .. * * .. * :. .::*:: *

XP_0120533 --SQVNFFLYLLLSSLDLTMGP-------ARSRLLSLYPLLS--------RATSLCLRAA

Homo_sapie NKAVIRYLPWLYHPPSAMQQGPKEFIECVSHIRLLSWLLLGSLTHNAVCPNASSPCLPIP

: :.:: :* .. : ** :: **** * * .*:* ** .

XP_0120533 SDYMS----------LQDVEGHGTFLISL-----AFMVMRLALLAVRDGPVSNEDREKGA

Homo_sapie LDAGSHVADHLIVILIGFPEQSKTSVLHMCSLFHAFIFAQLWTVYCEQSAVATNLQNQNE

* * : * * :: : **:. :* : .:..*:.: :::.

XP_0120533 GDEDAVDI---LWTRIWPEWIRLF---RLSFDPNCVN-----GALRTVTHSVFLDLLTFL

Homo_sapie FSFTAILTALEFWSRVTPSILQLMAHNKVMVEMVCLHVISLMEALQECNSTIFVKLIP--

. *: :*:*: *. ::*: :: .: *:: **: . ::*:.*:.

XP_0120533 GGIYSPILLRHTESLSVNLAMLVQY-----------------QETLGVAPSGKLQKATQI

Homo_sapie --MWLPMIQSNIKHLSAGLQLRLQAIQNHVNHHSLRTLPGSGQSSAGLAALRKWLQCTQF

:: *:: : : **..* : :* *.: *:*. * :.**:

XP_0120533 -MEKVGTHSVGGVADRMGMMESLKADLLAMERMKILNK

Homo_sapie KMAQVEIQSSEAASQFYPL-------------------

* :* :* ..:: :
